# Changes in Resveratrol Containing Phytosterol Liposomes During Model Heating

**DOI:** 10.3390/molecules30234645

**Published:** 2025-12-03

**Authors:** Joanna Igielska-Kalwat, Magdalena Rudzińska, Anna Grygier, Dominik Kmiecik, Katarzyna Cieślik-Boczula, Jolanta Tomaszewska-Gras

**Affiliations:** 1 Faculty of Food Science and Nutrition, Poznań University of Life Sciences, Wojska Polskiego 28, 60-637 Poznań, Poland; anna.grygier@up.poznan.pl (A.G.); dominik.kmiecik@up.poznan.pl (D.K.); jolanta.tomaszewska-gras@up.poznan.pl (J.T.-G.); 2 Faculty of Chemistry, University of Wroclaw, F. Joliot-Curie 14, 50-383 Wrocław, Poland; katarzyna.cieslik-boczula@uwr.edu.pl

**Keywords:** liposomes, resveratrol, oxidation, stability, phytosterols, heating system

## Abstract

Background: Phytosterols are bioactive lipids susceptible to oxidation, particularly under thermal stress. Incorporation into liposomes may enhance their stability, while resveratrol—a natural antioxidant—could further limit thermal degradation. Stigmasterol esters, which contain fatty acid residues prone to oxidation, require additional characterization to understand their behavior under heating. Methods: Liposomes composed of dipalmitoylphosphatidylcholine (DPPC) were enriched with free stigmasterol (ST), stigmasteryl myristate (ME), or stigmasteryl oleate (OE), with or without resveratrol (RES). Liposomal systems were characterized using transmission electron microscopy, zeta potential, and hydrodynamic diameter analyses. Samples were heated at 60 °C and 180 °C for 8 h to evaluate stigmasterol degradation, oxyphytosterol (SOP) formation, and decomposition of fatty acid residues in the esters. Results: Liposomes remained structurally stable at 60 °C but underwent marked alterations at 180 °C. ST formed the smallest particles, while ME and OE systems exhibited larger hydrodynamic diameters. Incorporation of resveratrol enhanced thermal and oxidative stability, reducing stigmasterol degradation (7.73–18.86% at 60 °C; 29.66–35.28% at 180 °C) and limiting SOP formation. Differences in the breakdown of myristic versus oleic acid residues highlighted the role of fatty acid type in determining thermal resistance. Conclusions: Resveratrol effectively improves the stability of liposomes containing stigmasterol or its esters and mitigates oxidative damage under thermal stress. Protective effects were particularly evident at moderate temperatures, indicating the potential of resveratrol–phytosterol liposomes as thermally stable delivery systems.

## 1. Introduction

**Figure 1:**
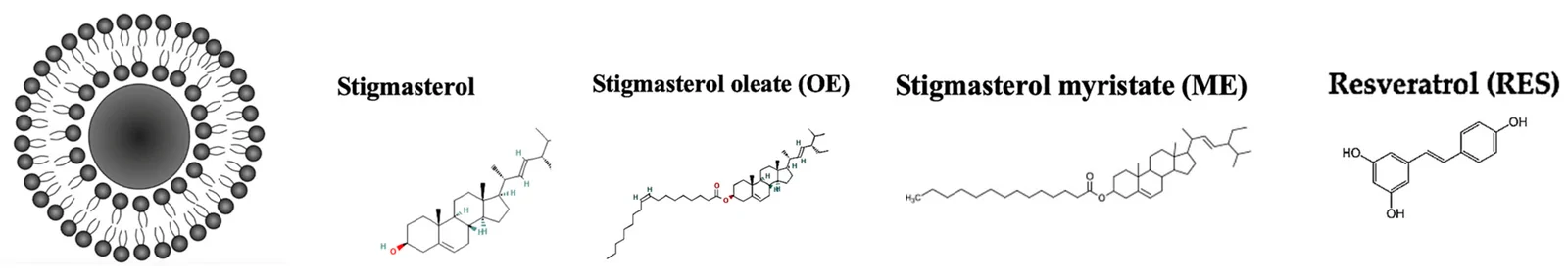
Chemical structure of the experimental resveratrol-containing liposomes.

Lipid nanocarriers offer significant potential for the encapsulation and delivery of bioactive compounds in the human body. Liposomes composed of dipalmitoylphosphatidylcholine (DPPC) with stigmasterol (ST), its esters (ME, OE), and resveratrol (RES) are self-assembled vesicular systems characterized by a lipid bilayer structure, making them an effective platform for phytosterol encapsulation in the food industry. These nanocarriers provide numerous advantages, including biocompatibility, high encapsulation efficiency, storage stability, enhanced bioavailability, targeted delivery, and prolonged release. The lipid nanocarriers we tested are capable of co-encapsulating both hydrophilic and hydrophobic compounds, enabling the synergistic delivery of bioactive substances [1,2,3]. Owing to their vesicular organization based on a phospholipid bilayer, liposomes can accommodate hydrophilic molecules within their aqueous core and hydrophobic compounds within the lipid membrane, which allows them to protect sensitive substances and modulate their stability. Phytosterols, including plant sterols and stanols (as well as their esters with fatty acids), are widely used in the medical, food, pharmaceutical, and cosmetic industries. These compounds offer various health benefits, including lowering total and LDL cholesterol levels in the blood. A daily intake of 2–3 g of phytosterols reduces total cholesterol by approximately 10% and LDL cholesterol by about 15%, thereby decreasing the risk of heart attack and preventing the development of atherosclerosis and ischemic heart disease [4]. They also promote vasodilation, improve circulation, and increase the permeability of blood vessel walls. Studies have shown that regular intake of phytosterols inhibits the formation of atherosclerotic plaques in coronary arteries [5,6]. Additionally, phytosterols modulate immune responses by suppressing allergic and autoimmune reactions, reducing lymphocyte counts, and inhibiting lymphoid tissue development [7,8,9,10,11,12,13]. However, the exact extent of their absorption in the human body remains unclear [14], with gastrointestinal absorption estimated at approximately 10%—significantly lower than that of cholesterol at 50–60% [15]. The presence of double bonds in their chemical structure makes phytosterols susceptible to thermal and oxidative degradation, leading to the formation of volatile compounds, oligomers, and biologically significant oxidation products known as oxysterols. These degradation products have been shown to be mutagenic, carcinogenic, cytotoxic, and immunosuppressive [16,17,18]. Because food products containing phytosterols and their esters are commonly subjected to heat treatment during cooking, baking, or frying, it is crucial to develop a delivery system that reduces the required doses and inhibits oxidation. Resveratrol (RES) is a natural polyphenolic compound with exceptional antioxidant activity, exceeding that even of vitamins C and E under certain conditions [19]. It has also been shown to modulate lipid metabolism, inhibit LDL oxidation, and prevent platelet aggregation [20,21,22]. As a phytoestrogen, resveratrol exhibits cardioprotective effects [23] as well as anti-inflammatory and anticancer properties [24]. To date, only a limited number of publications have explored the use of lipid carriers for phytosterols. One innovative approach involves incorporating resveratrol into the lipid bilayer in order to enhance the thermal stability, oxidative stability, and bioavailability of phytosterols. Phytosterols and their esters are functional ingredients in food formulations such as margarine, yogurt, and fruit and vegetable juices. These compounds are recommended for dietary use and are actively sought after by consumers [25]. The liposomal system we have tested, owing to its very small diameter and high bilayer membrane rigidity, represents a promising platform for the delivery of compounds with high bioavailability and stability. Furthermore, the combination of resveratrol and phytosterols fits the definition of functional food established by the European FUFOSE initiative, which includes antioxidants as natural food additives [26,27]. Health and food safety are of utmost importance to consumers: It is therefore essential to develop a safe delivery system for phytosterols that prevents thermal and oxidative degradation and enhances intestinal absorption. This would reduce the amount of phytosterols added to food without compromising their cholesterol-lowering effect, while also limiting the formation of harmful oxidation products and improving overall safety. The aim of this study is to develop an encapsulation procedure for phytosterols and their esters in resveratrol-enriched liposomes in order to improve their bioavailability and thermal and oxidative stability. In addition, particular attention was given to the degradation of fatty acid residues in stigmasterol esters, since these moieties may undergo oxidation during heat treatment and significantly influence the physicochemical integrity of the lipid bilayer. Because stigmasterol esters contain fatty acid residues, their degradation may directly influence the integrity and oxidative stability of liposomes. Therefore, in addition to assessing stigmasterol degradation, the thermal stability of the esterified fatty acid moieties (myristic and oleic acids) was also examined to better understand their contribution to liposomal stability and oxidative resistance. Figure 1 shows the chemical structure of the investigated liposomes containing resveratrol.

Hydrophobic components; stigmasterol, its esters (ME, OE), and resveratrol are located within the lipid bilayer, positioned between the acyl chains of DPPC. The aqueous central cavity of the liposome corresponds to the hydrophilic core. The DPPC bilayer forms the outer and inner membrane layers, serving as the structural matrix in which sterols and resveratrol are incorporated.

## 2. Results and Discussion

### 2.1. Phase Transition Analysis

**Figure 2:**
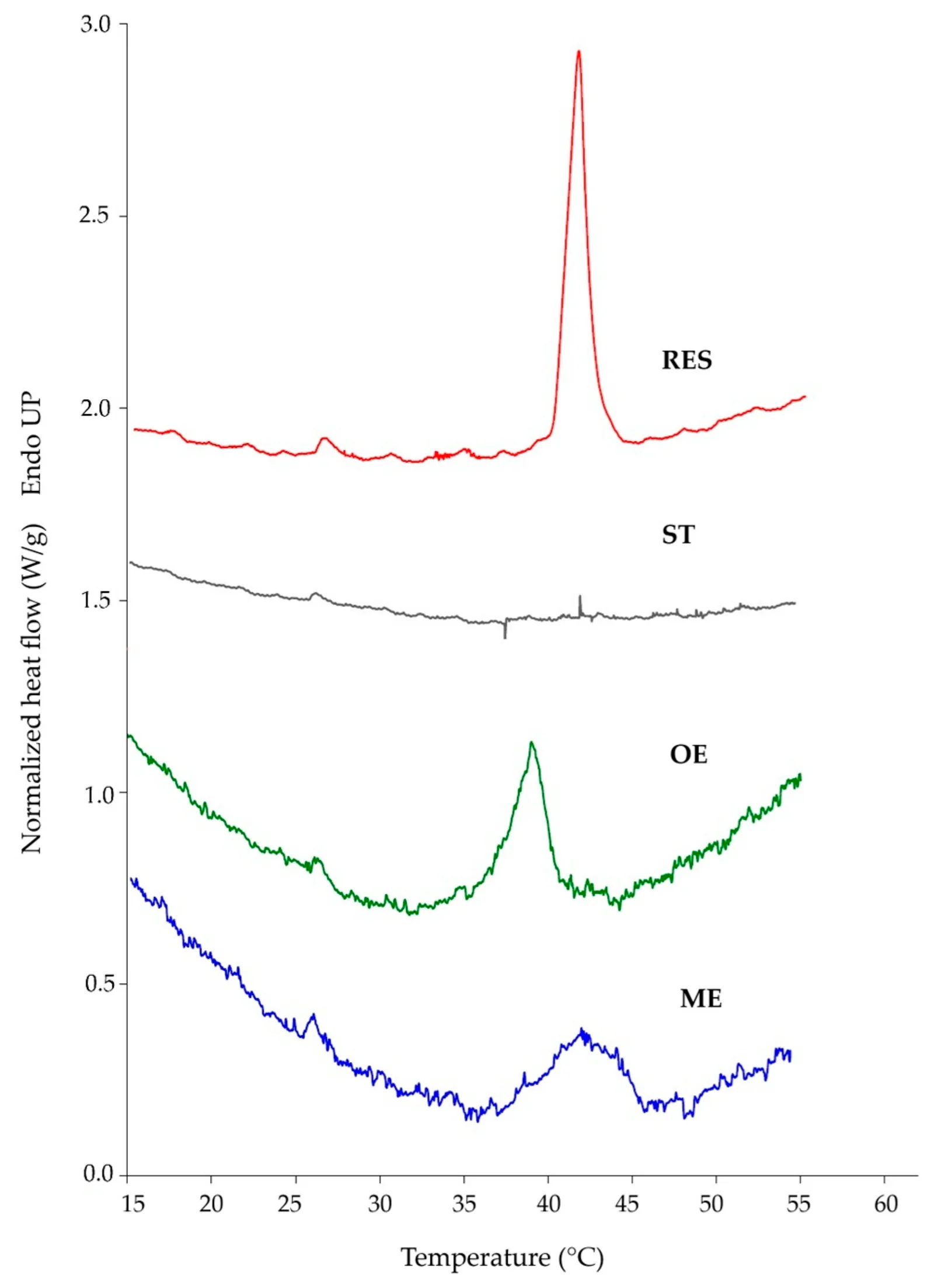
DSC melting phase transition curves of resveratrol-enriched DPPC liposomes (RES) and resveratrol-enriched DPPC liposomes with free stigmasterol (ST), stigmasteryl oleate (OE), and stigmasteryl myristate (ME).

The DSC technique was used to measure the thermotropic parameters of phase transition of the DPPC liposomes prepared with resveratrol and resveratrol-enriched DPPC liposomes with stigmasterol, stigmasteryl oleate and stigmasteryl myristate. In Figure 2 the DSC melting curves of liposome samples are shown, and in Table 1 parameters calculated from phase transition profiles such as onset temperature (T_on_), peak temperature (T_p_), width at half height (ΔT_1/2_) and enthalpy (∆H). The DSC curve of DPPC liposomes with resveratrol showed an endothermic peak with a maximum (T_p_) at 41.39 °C (Table 1), which corresponded to the main phase transition. It can also be noticed that the transition was very sharp with an onset temperature (T_on_) 40.17 °C and a peak half-width of ΔT_1/2_ = 1.24 °C, which parameter is often used as a measure of the cooperativity of the transition. These results are consistent with a previous study on the DPPC, where for pure liposomes the melting phase transition was measured at 42 °C [28]. Similarly, the values of enthalpy (44.24 J/g) and response ratio (0.724 mW/°C) are the highest for the RES sample, indicating the integration of the DPPC bilayer. It is worth mentioning that resveratrol is a hydrophobic compound, sparingly soluble in water, and it incorporates into the lipid bilayer similarly to ST and its derivatives (OE, ME). Figure 2 shows that the incorporation of ST, OE, and ME caused changes in the bilayer, as seen in the altered curves of phase transition. In the case of ST, no peak corresponding to the phase transition was observed, whereas for OE and ME, the peak broadened and its height decreased. All of these phenomena were reflected in the thermotropic parameters of OE and ME during the melting phase transition, compared to the RES sample. Both esters exhibited higher peak half-widths (2.13 and 4.51 °C for OE and ME, respectively) and lower enthalpy values (32.43 and 28.71 J/g), as well as reduced response ratios (0.109 and 0.0315 mW/°C, respectively). The position of the peak was also shifted; however, the peak temperature was lower for OE (39.46 °C) and higher for ME (42.35 °C) compared to RES (41.39 °C), which can be explained by the different melting points for oleic acid (16 °C) and myristic acid (55 °C). This observation may indicate that the incorporation of ME into the DPPC bilayer increases the melting temperature of liposomes, making them more stable than pure DPPC liposomes. An opposite effect was observed for OE, which lowered the melting temperature of DPPC liposomes. The results obtained suggest that the incorporation of pure ST or its esters OE and ME had influence on the integrity of the DPPC bilayers, which was observed by the broadening of the main phase transition peak, for which the onset temperature (T_on_) was shifted to lower values from 40.17 °C for RES to 39.46 °C and 42.35 °C, respectively, indicating increasing structural disorder in the bilayer. Similar effects of the loss of cooperativity of the observed melting phase transitions were observed in the studies on the incorporation of α-tocopherol and its derivatives into DPPC liposomes [29].

### 2.2. Zeta Potential and Hydrodynamic Diameter of the Liposomes

The zeta potential reflects the surface charge of a particle, which affects its stability. The zeta potential of DPPC liposomes containing resveratrol in the presence of ST, ME, and OE is presented in Table 2. Liposomes formulated with ST exhibited a slightly positive zeta potential (2.9 mV), which is characteristic of DPPC-based systems [30]. In formulations containing ME, a further increase in zeta potential was observed, while liposomes with OE showed a reduction in this parameter, reaching 3.1 mV and 2.0 mV, respectively [31]. Studies in the literature have also confirmed that ergosterol and its esters with oleic, linoleic, and linolenic acids shift the zeta potential of egg phosphatidylcholine liposomes in the negative direction by approximately 2–5 mV [32]. In our study, this phenomenon may result from stronger interactions (than in the case of free stigmasterol) of stigmasterol esters with the hydrophobic part of DPPC liposomes—a direct consequence of their increased lipophilicity. This may contribute to the disruption of the DPPC gel phase and, consequently, to the reorganization of phospholipid head groups. This is supported by the greater change in zeta potential seen for OE than for ME. The oleic acid residues in OE, on account of their cis conformation, have a bent hydrocarbon chain, which increases membrane fluidity and brings its structure closer to the liquid-crystalline state. It is also worth noting that the relatively low positive zeta potential values of DPPC in the presence of stigmasterol and its esters do not favor liposome stability. In the present study, the uniformity of the liposomal populations was assessed based on the variability of the hydrodynamic diameter (mean ± SD), which reflects the width of the particle size distribution. The low standard deviation values observed for the ST + RES liposomes indicate a more homogeneous system, whereas the higher SD values recorded for the ME + RES and OE + RES formulations suggest greater polydispersity, which is typically associated with reduced physical stability. The variability of the diameters and the corresponding SD values provide an indirect measure of the distribution width and allow for an estimation of the polydispersity of the analyzed liposomes. The final size of the liposomes is also strongly dependent on the packing efficiency of the lipid molecules during vesicle formation and their phase state relative to the melting point (Tm) of the incorporated sterol esters. Liposomes produced at temperatures below the Tm of the esterified sterol typically exhibit reduced molecular mobility within the bilayer, leading to imperfect packing and the formation of larger vesicles. This mechanism is consistent with the markedly higher sizes observed for the ME + RES and OE + RES samples (644 nm and 945 nm, respectively), compared with ST + RES (214 nm). The bent conformation of oleic acid in OE further disrupts chain packing and enhances membrane fluidity, promoting even larger vesicle formation. These observations align with our DSC results, which showed broadened and less cooperative phase transitions in ester-containing formulations, indicating altered packing and increased disorder within the bilayer. Overall, these findings demonstrate that differences in molecular organization and phase behavior during membrane formation play a key role in determining the final liposome size.

Significant variations in the zeta potential (ZP) of the liposomes were observed after exposure to a temperature of 60 °C (Table 2). In the liposomes containing free stigmasterol, a marked decrease in ZP was noted, from 2.9 mV to −15.0 mV. This shift may be attributed to the increased membrane fluidity induced by oxidation products. In contrast, the changes observed for ME + RES and OE + RES were considerably milder (8.3 mV and 4.7 mV, respectively), suggesting that stigmasterol esters may offer partial protection against peroxidation-induced membrane disturbances. Upon heating to 180 °C, all samples showed a pronounced shift in zeta potential toward highly negative values (−41.2 mV, −38.5 mV, and −33.5 mV for ST + RES, ME + RES, and OE + RES, respectively). These findings indicate that neither free nor esterified phytosterols were able to protect the membrane from severe structural alterations under such intense oxidative stress. A key parameter used to characterize liposomes is their hydrodynamic diameter (Table 1): In this experiment, significantly greater hydrodynamic diameters were observed in the presence of stigmasterol esters than in the free form: 644 nm for ME + RES and 945 nm for OE + RES, versus 214.2 nm for ST + RES. The study of Rudzińska et al. [30] indicates that phytosterols contribute to an increase in the size of multilamellar DPPC liposomes. They also reported that the presence of free phytosterols tends to increase liposome diameter, whereas the incorporation of phytosterols esters may lead to a decrease. Those authors suggest that ester molecules can partially replace phospholipids within the membrane structure or form lipid domains, resulting in tighter lipid packing. This phenomenon is primarily observed in membranes composed of natural phospholipids, where differences in hydrocarbon chain lengths, saturation levels, and polar head group structures make perfect packing difficult [30,31,32]. In the case of DPPC-based liposomes, which are highly ordered, the presence of phytosterols has been shown to cause membrane expansion through interactions among lipid chains, the creation of interlipid spaces, and membrane reorganization [30,31,32]. In this study, similar mechanisms may explain the increase in particle size, particularly in the presence of OE (945 nm), which could result from enhanced membrane fluidity due to the bent oleic acid residues. Upon heating the liposomes to 60 °C and 180 °C, an increase in hydrodynamic diameter was observed for ST + RES (up to 556.6 nm at 180 °C), while a reduction was noted for ME + RES (to 244.8 nm) and for OE + RES (to 188.6 nm at 180 °C). These size changes may be attributed to differences in the susceptibility of free and esterified phytosterols to oxidation, which in turn may disrupt the hydrophilic–lipophilic balance of the membrane. In addition to oxidation-driven alterations, the differences in particle size can also be interpreted in the context of molecular packing within the lipid bilayer. The incorporation of stigmasterol esters into DPPC affects membrane order in a manner directly related to the melting temperature of the fatty acid moiety. Since the liposomes in our study were formed below the melting point of stigmasteryl myristate (55 °C) and well above the melting point of stigmasteryl oleate (16 °C), the packing state of these molecules differed substantially during vesicle formation. ME, being in a more ordered state during hydration and sonication, promotes tighter packing and reduced membrane fluidity, which can hinder vesicle division and favor the formation of larger multilamellar structures. In contrast, OE liquid at the liposome preparation temperature, induces disorder in the bilayer, facilitating curvature formation and leading to smaller, more flexible vesicles. These packing-dependent effects provide a complementary explanation for the observed differences in hydrodynamic diameter, supporting our hypothesis that both thermal behavior and structural organization of the esterified molecules contribute to the final size and stability of the liposomes.

### 2.3. Stability of ST, ME, and OE Liposomes with RES

#### 2.3.1. Stigmasterol Degradation

**Figure 3:**
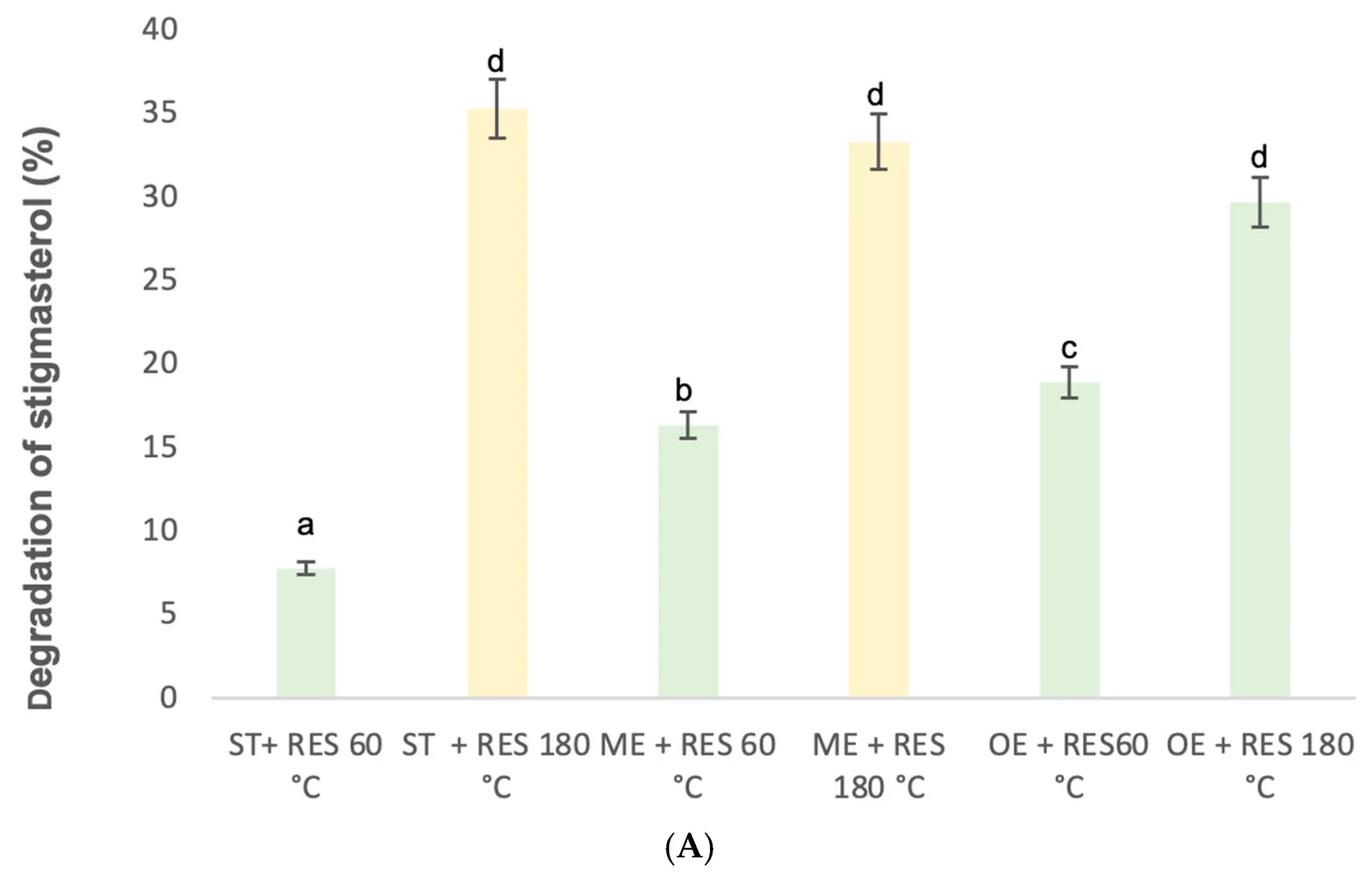

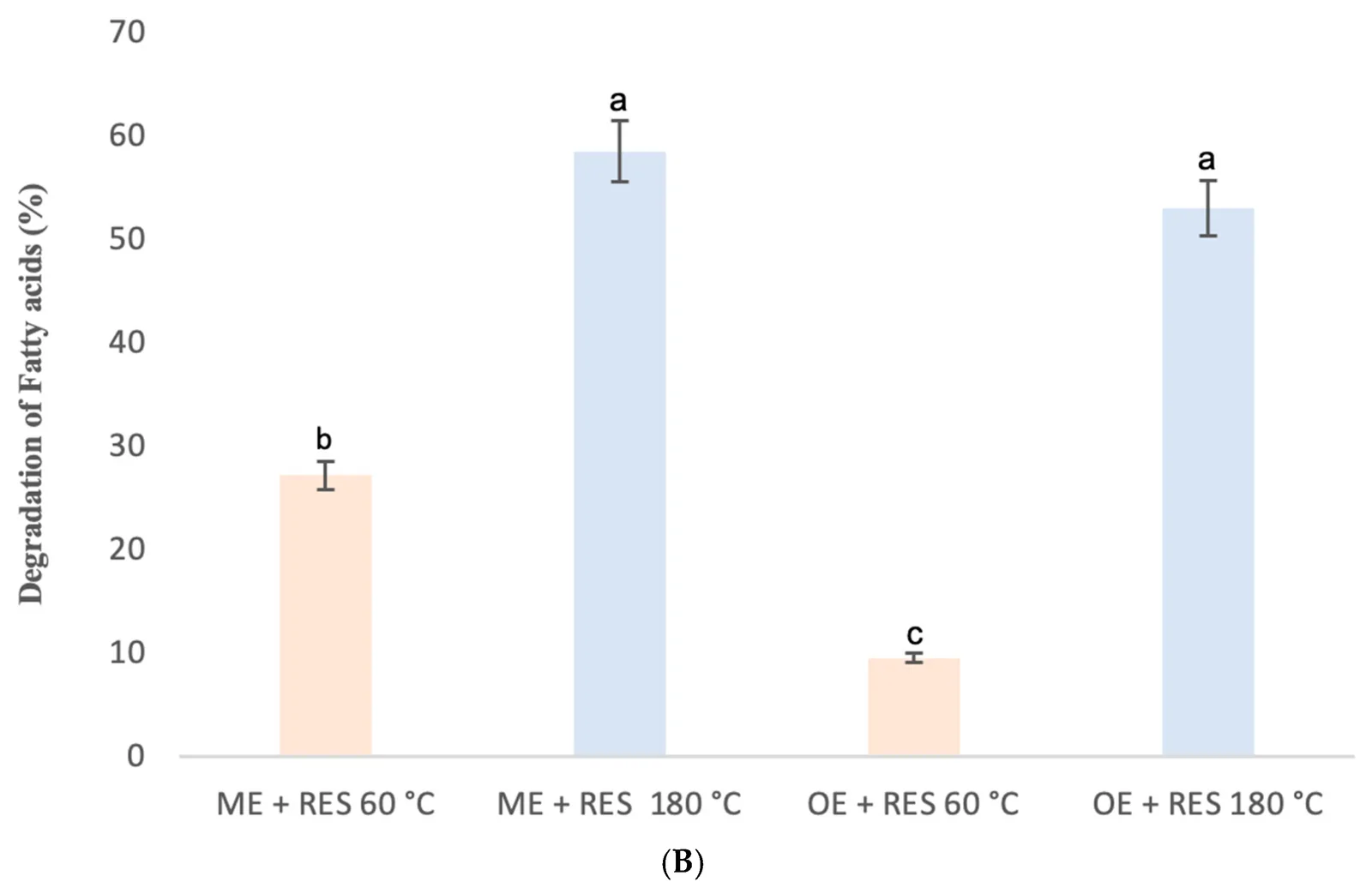
Degradation of (**A**) stigmasterol and (**B**) fatty acids after thermo-oxidation of liposomes containing resveratrol enriched with free stigmasterol (ST + RES), myristic ester of stigmasterol (ME + RES), and oleic ester of stigmasterol (OE + RES) held at 60 °C and 180 °C for eight hours. Values represent mean ± standard deviation (*n* = 3). Bars marked with different superscript letters indicate statistically significant differences among treatments (*p* < 0.05; one-way ANOVA followed by Tukey’s post hoc test).

Liposomes containing resveratrol were enriched with three different compounds: free stigmasterol, stigmasteryl myristate, and stigmasteryl oleate. The choice of stigmasterol was motivated by its chemical structure: it contains two double bonds, which may make it susceptible to oxidation at elevated temperatures. Additionally, the availability of a pure synthetic standard of this compound, free from other phytosterols, was a significant advantage. The extent of stigmasterol loss in liposomes heated in the presence of resveratrol depended on its chemical form (free or esterified), the type of fatty acid used for esterification, and the temperature. When heated to 60 °C, the degradation level of stigmasterol was 7.73% for ST + RES, 16.31% for ME + RES, and 18.86% for OE + RES (Figure 3A). After exposure to 180 °C, the lowest loss was observed in the OE + RES sample (29.66%), while degradation reached as high as 35.38% and 33.27% for ST + RES and ME + RES, respectively. This indicates that the free form was more resistant at lower temperatures, and that the degree of degradation increased with more intense heating. Interestingly, the degradation level remained similar regardless of temperature, suggesting that oleic acid may exert a stabilizing effect on stigmasterol. For a better understanding of the protective role of resveratrol, the antioxidant activity of liposomes with and without resveratrol was also compared, and the corresponding results are presented in Figure 3A

Resveratrol contributes to enhanced stability and reduced degradation of stigmasterol in liposomes composed of DPPC (dipalmitoylphosphatidylcholine) primarily on account of its potent antioxidant properties. The incorporation of resveratrol, a natural polyphenol, into the liposome structure stabilizes the lipid bilayer in two ways: First, resveratrol limits stigmasterol oxidation by neutralizing free radicals that initiate phytosterols degradation, leading to a decrease in the thermal degradation rate of the phytosterols. Second, by modifying the physicochemical properties of the membrane, resveratrol can integrate into the DPPC bilayer, increasing its order and rigidity. This restricts the penetration of oxygen molecules and free radicals into the membrane interior, further protecting the encapsulated bioactive phytosterols against oxidation. Our results indicate that the incorporation of resveratrol into liposomes effectively increased stigmasterol resistance to thermally induced degradation [33,34].

#### 2.3.2. Degradation of Fatty Acids

The degradation rate of the myristic acid residue during thermal treatment was 27.17% at 60 °C and 58.49% at 180 °C, which was higher than the level of degradation of the oleic acid residue (Figure 3B). For oleic acid, the loss was 9.50% at 60 °C and 52.99% at 180 °C. Despite having a double bond in its structure, oleic acid underwent less degradation than myristic acid, indicating its greater thermal stability. These findings from resveratrol-enriched liposomes are consistent with previous reports on conventional liposomal systems. It has been shown that liposomes containing oleic acid exhibit enhanced resistance to lipid oxidation and maintain structural integrity during long-term storage [34]. Our data suggest that the thermal stability of fatty acids differs with where they are located, for example, as part of the phospholipid bilayer or encapsulated within the liposome. In this context, oleic acid, when incorporated as a phytosterols ester, offered more effective protection against stigmasterol degradation than did myristic acid. Our results show that the stability of fatty acids enclosed in liposomes containing stigmasteryl oleate and supplemented with resveratrol exhibited the most effective protective properties. Moreover, resveratrol significantly enhances liposome stability by acting on multiple levels. By being incorporated into the phospholipid bilayer structure, it increases membrane order and rigidity, which limits the penetration of oxygen and free radicals. As a result, the susceptibility of liposomes to degradation is reduced, especially during heating. Additionally, resveratrol affects the hydrophilic–lipophilic balance of the system, supporting the long-term physical stability of the liposomes [34].

#### 2.3.3. Stigmasterol Oxidation Products

**Figure 4:**
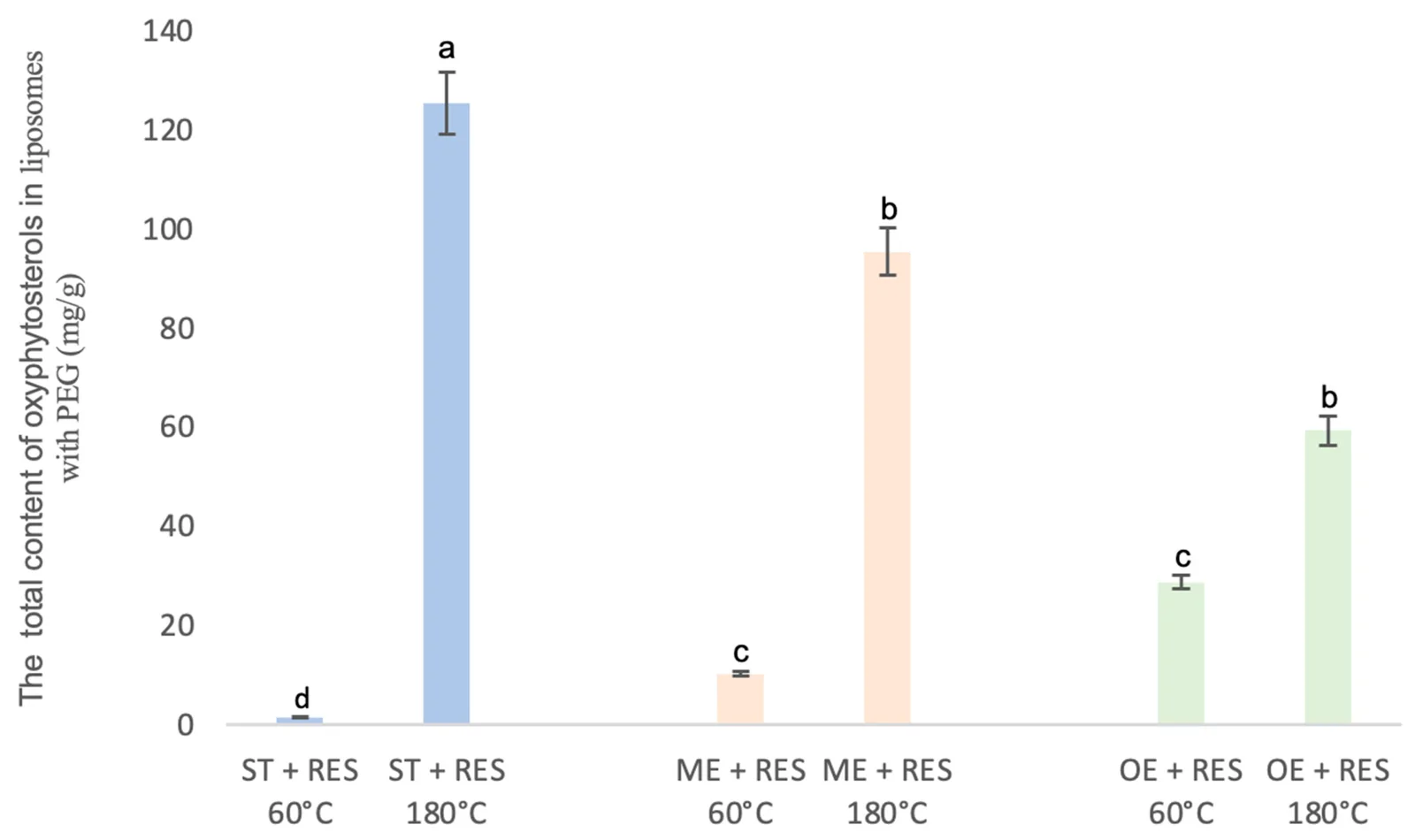
SOPs formed during holding of liposomes with resveratrol enriched with free stigmasterol (ST + RES), myristic ester of stigmasterol (ME + RES), and oleic ester of stigmasterol (OE + RES) at 60 °C and 180 °C for eight hours. Values represent mean ± standard deviation (*n* = 3). Bars marked with different superscript letters indicate statistically significant differences among treatments (*p* < 0.05; one-way ANOVA followed by Tukey’s post hoc test). SOP levels are expressed as mg of stigmasterol oxidation products per gram of stigmasterol (mg/g).

The level of phytosterols oxidation products (SOPs) in heated liposomes with resveratrol, containing free stigmasterol and its esters, is presented in Figure 4. SOPs were not identified in the liposomes prior to heating.

Analysis showed that the concentration of stigmasterol oxidation products (SOPs) in liposomes heated to 60 °C ranged from 1.56 mg/g in the samples with free stigmasterol to 10.36 mg/g and 28.83 mg/g in those containing stigmasteryl myristate and oleate, respectively. When subjected to a higher temperature of 180 °C, the total SOP content increased significantly, reaching 59.38 mg/g for oleate-based liposomes and rising to 95.54 mg/g and 125.63 mg/g in liposomes with stigmasteryl myristate and free stigmasterol. Incorporating resveratrol into these formulations led to a noticeable reduction in the accumulation of harmful oxysterols, as compared to conventional liposomal preparations [34]. This reduction was most likely due to the chemical structure of resveratrol, which effectively neutralizes reactive oxygen species (ROS), key initiators of the autoxidation process of phytosterols, including stigmasterol. Resveratrol integrates into the lipid bilayer, increasing membrane rigidity, reducing its permeability to oxygen and other oxidizing agents, and thus enhancing the thermal and oxidative stability of the entire liposomal system. As a result, the oxidation of phytosterols within the liposomal membrane is significantly minimized. Liposomes containing free stigmasterol and resveratrol demonstrated the greatest oxidative stability under simulated accelerated storage conditions (60 °C). It is important to note that DPPC itself does not exhibit chemical antioxidant activity; the reduced oxidation observed in DPPC-based liposomes arises primarily from a physical barrier effect of the tightly packed gel phase, which limits oxygen permeation and restricts free radical diffusion within the bilayer. Resveratrol additionally exhibits metal-chelating properties that enable it to bind metal ions such as Fe^2+^ and Cu^2+^; these serve as cofactors in the Fenton reaction, a major source of hydroxyl radicals, and are among the most potent oxidants known. The outcome is the inhibition of lipid and phytosterol oxidation cascades initiated by metal-catalyzed reactions. The autoxidation of phytosterols typically begins with hydrogen abstraction at the C7 allylic position within the B ring of the molecule, leading to the formation of 7α- and 7β-hydroperoxides. These peroxides subsequently produce hydroxysterols and can undergo further oxidation to form 7-ketosterol. An alternative oxidative pathway targets the Δ5,6 double bond, yielding α- and β-epoxide intermediates, which may be metabolized to 3β,5α,6β-triol [35]. Six distinct SOPs were identified in the thermally treated liposomes, and their profiles varied by formulation. In samples heated to 60 °C, epoxide derivatives were dominant in the ST sample, whereas the ME liposomes exhibited the highest levels of both 7-hydroxystigmasterol isomers. For the OE liposomes, α-configured derivatives such as 7αOHSt and α-epoxySt were most abundant [36,37]. All liposomes exposed to 180 °C demonstrated increased α-epoxySt concentrations, with ester-based liposomes also producing β-epoxySt. Liposomes containing free stigmasterol and resveratrol showed the greatest oxidative stability, while esterifying stigmasterol with myristic or oleic acid decreased resistance to oxidation. At 180 °C, α-epoxystigmasterol and 7-ketostigmasterol dominated in the ST sample, whereas ester-based liposomes produced the highest levels of α-epoxySt, followed by β-epoxySt. The ME sample reached the highest triolSt concentration (3.05 mg/g at 60 °C; 10.53 mg/g at 180 °C), making it the variant most susceptible to deep oxidative transformations.

### 2.4. Antioxidant Activity of Liposomes

#### 2.4.1. Determination of Percentage Inhibition of ST, OE, and ME Liposomes as Well as of ST + RES, OE + RES, and ME + RES

**Figure 5:**
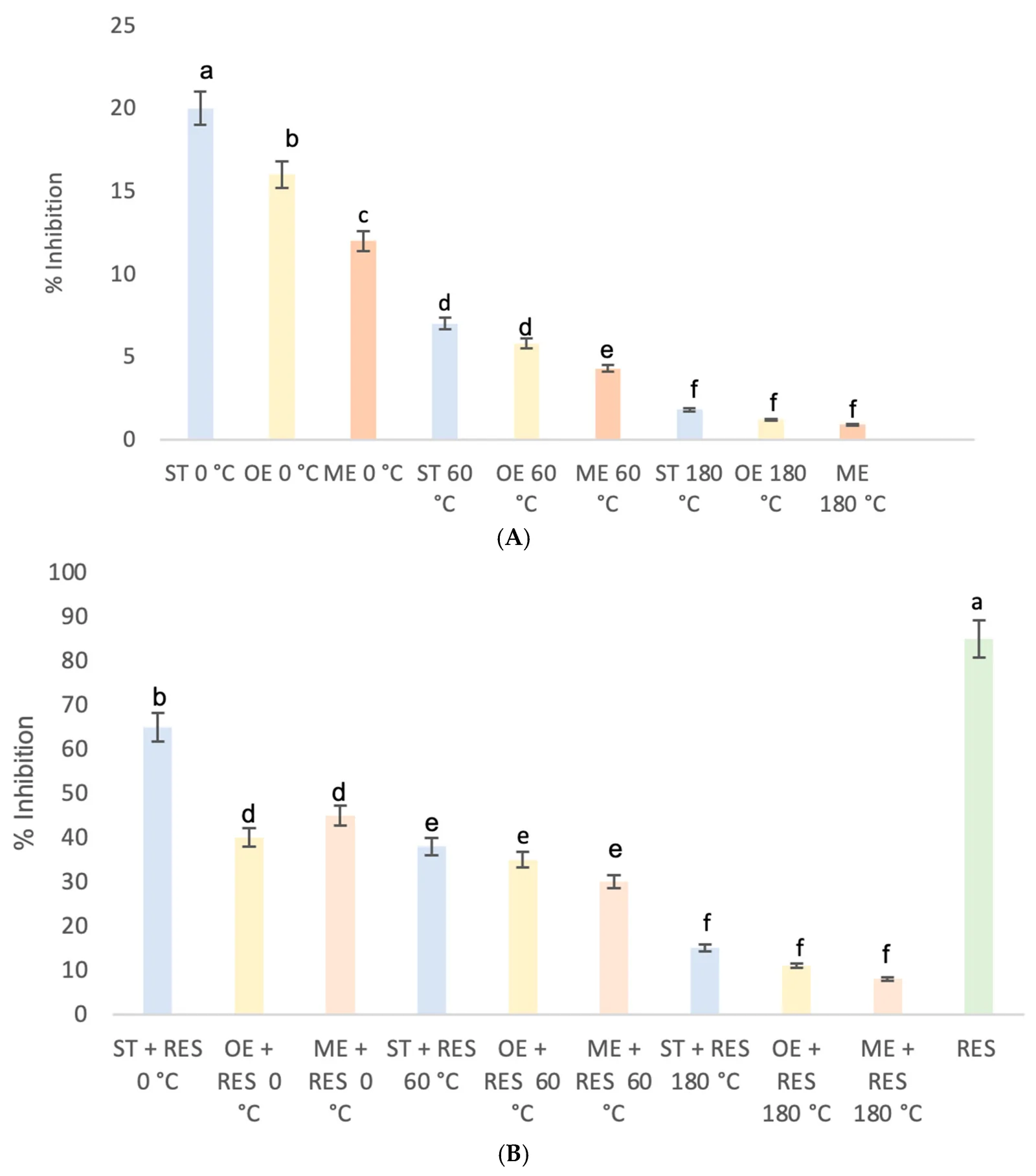
Antioxidant activity. (**A**). Conventional liposomes without resveratrol. (**B**). Liposomes containing resveratrol. Values are presented as mean ± SD (*n* = 3). Bars marked with different superscript letters indicate statistically significant differences among samples (*p* < 0.05; one-way ANOVA with Tukey’s post hoc test).

For comparison of the percentage inhibition of ST, OE, and ME liposomes with resveratrol, we examined a control sample consisting of liposomes with the same composition but lacking resveratrol. Figure 5 presents the antioxidant activity of both types.

As shown in Figure 5A, the highest percentage of inhibition was observed for the ST 0 °C liposome (20%), followed by OE 0 °C (16%); the least inhibition was seen for ME 0 °C (12%). Liposomes composed of dilinoleoylphosphatidylcholine (DPPC) with free stigmasterol demonstrated moderate inhibition, which can be attributed to the inherent antioxidant properties of DPPC. Moreover, free stigmasterol itself exhibits some antioxidant activity. DPPC liposomes containing stigmasteryl myristate showed lower levels of inhibition than did those containing stigmasteryl oleate. This may be due to the structural differences in the fatty acids: oleic acid (C18:1) contains a double bond, which can contribute to antioxidative effects, whereas myristic acid (C14:0) is saturated and lacks direct antioxidant activity, though it may help stabilize the liposomal membrane. Stigmasteryl oleate also improves the physicochemical properties of liposomes, which may enhance their antioxidant activity. Temperature had a significant influence on the outcomes [38]. At 60 °C, stigmasterol remains relatively stable, though minor oxidation may occur, reducing its antioxidative properties. Stigmasterol degrades at 180 °C, leading to the formation of products with minimal or no antioxidant activity. At 60 °C, increased membrane fluidity may enhance the accessibility of stigmasterol to DPPH radicals, allowing the liposomes to remain relatively stable. However, the liposomes degrade entirely at 180 °C: the phospholipids lose their integrity and undergo oxidation, resulting in the formation of lipid peroxidation products that may act as prooxidants instead of antioxidants. DPPC liposomes also become unstable and disintegrate at 180 °C. This relationship is visible in Figure 5A. The inhibition values for liposomes at 60 °C were: ST 60 °C (7%), OE 60 °C (5.8%), and ME 60 °C (4.3%). For liposomes heated to 180 °C, the inhibition values were: ST 180 °C (1.8%), OE 180 °C (1.2%), and ME 180 °C (0.9%). In the liposomes containing free stigmasterol (ST + RES 0 °C, ST + RES 60 °C, ST + RES 180 °C), stigmasteryl oleate (OE + RES 0 °C, OE + RES 60 °C, OE + RES 180 °C), and stigmasteryl myristate (ME + RES 0 °C, ME + RES 60 °C, ME + RES 180 °C), the same amount of resveratrol was added at a concentration of 5 mol%. Figure 5B presents the antioxidant activity of the liposomes enriched with resveratrol. Resveratrol’s percentage inhibition (its ability to scavenge DPPH radicals) depends on its concentration and on the experimental conditions. At low concentrations (10–25 µM), resveratrol exhibits moderate radical-scavenging activity. At higher concentrations (50–100 µM), its antioxidant capacity increases significantly, reaching nearly 100% inhibition. The concentration that causes 50% inhibition of DPPH**•** (the IC50 value) for resveratrol is typically within the range of 20–30 µM [39]. In Figure 5B, an increase in the percentage inhibition was observed for the liposomes that were not subjected to thermal treatment (ST + RES 0 °C, OE + RES 0 °C, and ME + RES 0 °C). The highest antioxidant activity level (65%) was shown by the ST + RES 0 °C liposomes. This is due to the fact that, as in the case of conventional liposomes, stigmasterol is a phytosterols containing a steroid ring and a side chain with a double bond. Stigmasterol esters (myristate and oleate) are derivatives in which the hydroxyl group at the C3 position has been esterified [40]. For antioxidant activity, this means that the free hydroxyl group (-OH) at C3 in stigmasterol can donate a proton and participate in reactions with the DPPH radical. However, esterification of this hydroxyl group (e.g., with myristic or oleic acid) blocks it, which may hinder direct proton donation to DPPH**•**. If the mechanism of DPPH**•** scavenging by stigmasterol is mainly based on the hydroxyl group, then esters may exhibit weaker antioxidant activity. Additionally, the esters (myristate and oleate) are more hydrophobic than stigmasterol because they contain long fatty acid chains. In tests with DPPH**•**, which are usually carried out in methanolic solutions, solubility can significantly affect the efficiency of interaction with the radical. Due to the fact that esters had poorer solubility in the test system, their ability to scavenge DPPH**•** was lower than that of free stigmasterol [41]. The OE + RES 0 °C liposome, with 50% inhibition, showed stronger activity than the ME + RES 0 °C liposome, with 40% inhibition. Liposomes containing stigmasteryl oleate will be better antioxidants than those containing stigmasteryl myristate. As previously mentioned, oleic acid (C18:1) contains a double bond, which can contribute to its antioxidant activity, while myristic acid (C14:0) is a saturated fatty acid that lacks the ability to scavenge free radicals. Myristic acid (C14:0) is more oxidatively stable and may provide a longer shelf-life for liposomes, but it does not enhance their antioxidant capacity. Its role is primarily limited to stabilizing the structure of liposomes [42]. It may be more resistant to oxidation, but that does not mean it acts as an antioxidant itself. In contrast, oleic acid can help neutralize free radicals: it acts as an antioxidant by helping to scavenge radicals and preventing lipid oxidation. It may also stabilize phospholipids in liposomes and enhance the antioxidant protection of the entire system [43]. Oleic acid (C18:1) is more prone to oxidation, though its presence increases the neutralizability of free radicals, which can be beneficial in the short term. The high level of antioxidant activity is due to the presence of resveratrol, which is a potent antioxidant that interrupts radical reactions by donating hydrogen atoms or electrons, leading to the formation of more stable compounds [43,44,45]. Resveratrol is a polyphenol that contains three hydroxyl (–OH•) groups. These groups can easily donate hydrogen atoms, which enables the neutralization of reactive oxygen species (ROS) and free radicals, thus preventing oxidative stress. Additionally, resveratrol is a stilbenoid and thus contains a system of conjugated double bonds, which helps stabilize the free radicals formed after electron donation [46]. As a result, it acts as an effective free radical scavenger. Resveratrol not only acts directly as an antioxidant, but also activates genes such as sirtuins (SIRT1), which protect against oxidative stress and support repair mechanisms [47].

The liposomes were next heated to 60 °C to assess their thermal stability under simulated storage conditions and to observe the decrease in percentage inhibition. In Figure 5B, a lower percentage inhibition was observed for liposomes subjected to heat treatment (ST + RES 60 °C, OE + RES 60 °C, and ME + RES 60 °C) than for those that were not heated. The percentage inhibition values of the liposomes were: 38% for ST + RES 60 °C, 35% for OE + RES 60 °C, and 30% for ME + RES 60 °C. Resveratrol begins to lose its stability at 60 °C, but retains some antioxidant activity. Liposomes were also raised to 180 °C to assess their thermal stability under simulated frying conditions. The lowest percentage inhibition values at 180 °C were observed for the ST + RES 180 °C, OE + RES 180 °C and ME + RES 180 °C (Figure 5B) systems, at 15%, 11%, and 8%, respectively. Pure resveratrol undergoes significant degradation at 180 °C, which reduces its ability to scavenge free radicals. Trans-resveratrol may isomerize to cis-resveratrol, which has weaker antioxidant activity, and may undergo oxidation and polymerization, leading to the formation of compounds with reduced DPPH**•** -scavenging capacity [48]. Stigmasterol is relatively stable at 60 °C, although slight oxidation may occur. However, oxidation and breakdown of the stigmasterol molecule occur at 180 °C, resulting in products with potentially diminished or even absent antioxidant activity. The effect of temperature on the structure of liposomes is very significant: increased membrane fluidity may occur at 60 °C, which can in fact improve the accessibility of resveratrol and stigmasterol to DPPH radicals; the liposomes remain relatively stable. In contrast, liposomes at 180 °C undergo complete degradation, while phospholipids lose their integrity and may oxidize; lipid peroxidation products form, which may act as pro-oxidants rather than antioxidants [49].

#### 2.4.2. Measurement of the Ability to Scavenge DPPH**•** (1,1-Diphenyl-2-picrylhydrazyl) Radical by Liposomes ST, OE, and ME, as Well as ST + RES, OE + RES, and ME + RES

**Figure 6:**
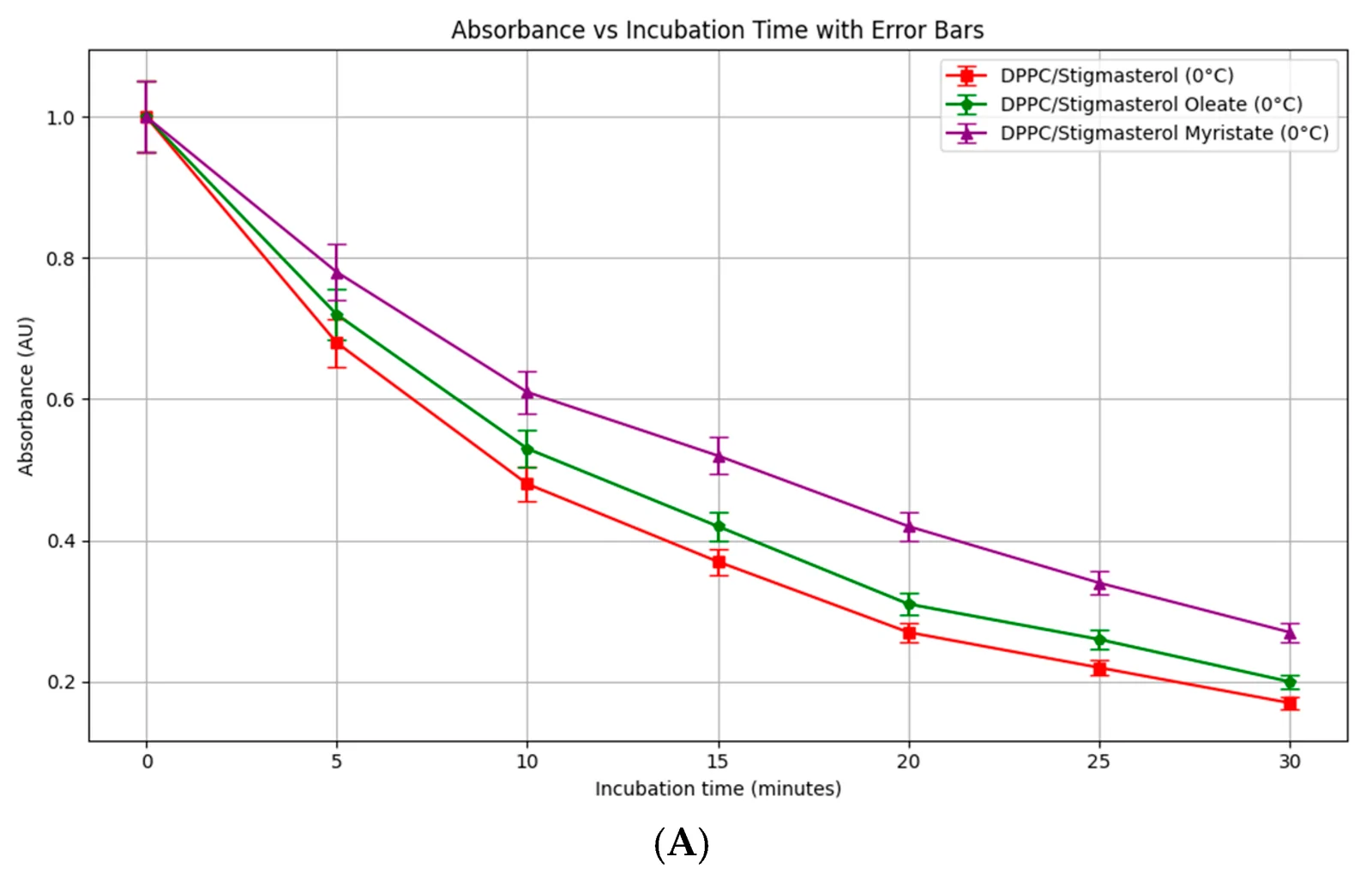

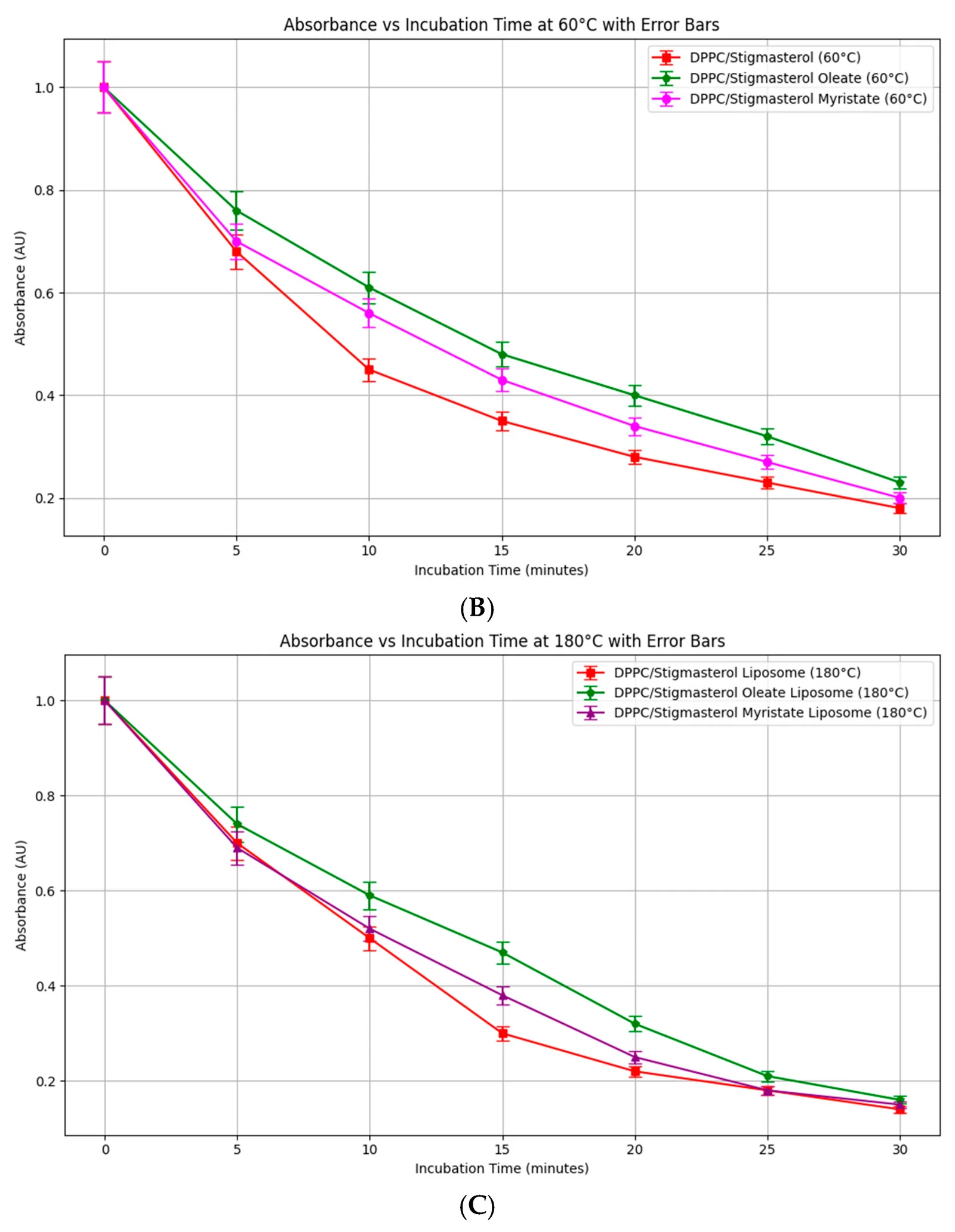
1,1-diphenyl-2-picrylhydrazyl free radical scavenging ability of liposomes containing free stigmasterol (ST) and its esters with oleic acid (OE) and myristic acid (ME), not enriched with resveratrol, held at 0 °C (**A**), 60 °C (**B**), and 180 °C (**C**).

Figure 6 shows measurements of the free radical scavenging ability of 1,1-diphenyl-2-picrylhydrazyl by liposomes containing free stigmasterol (ST) and its esters with oleic acid (OE) and myristic acid (ME) at different temperatures.

**Figure 7:**
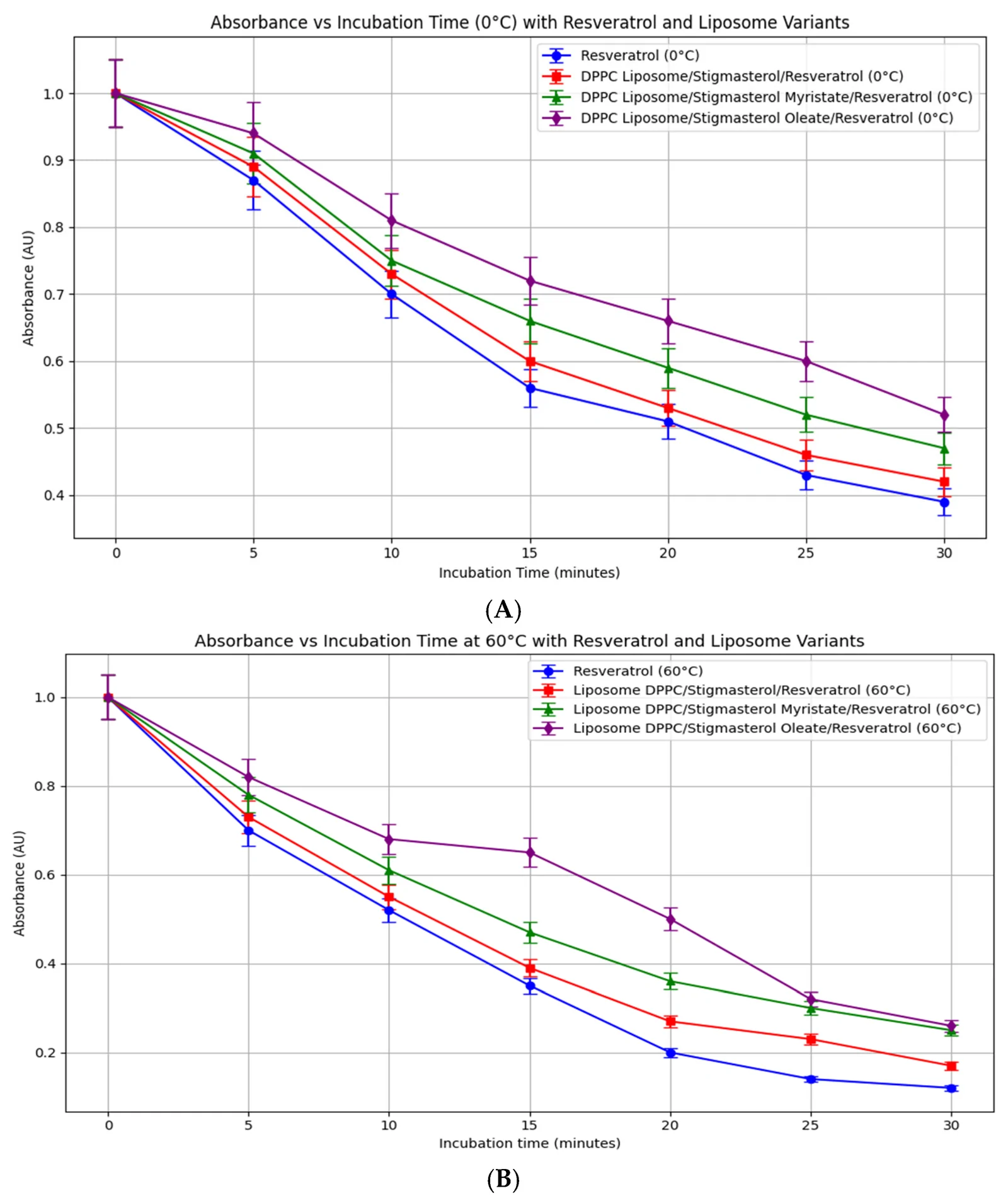

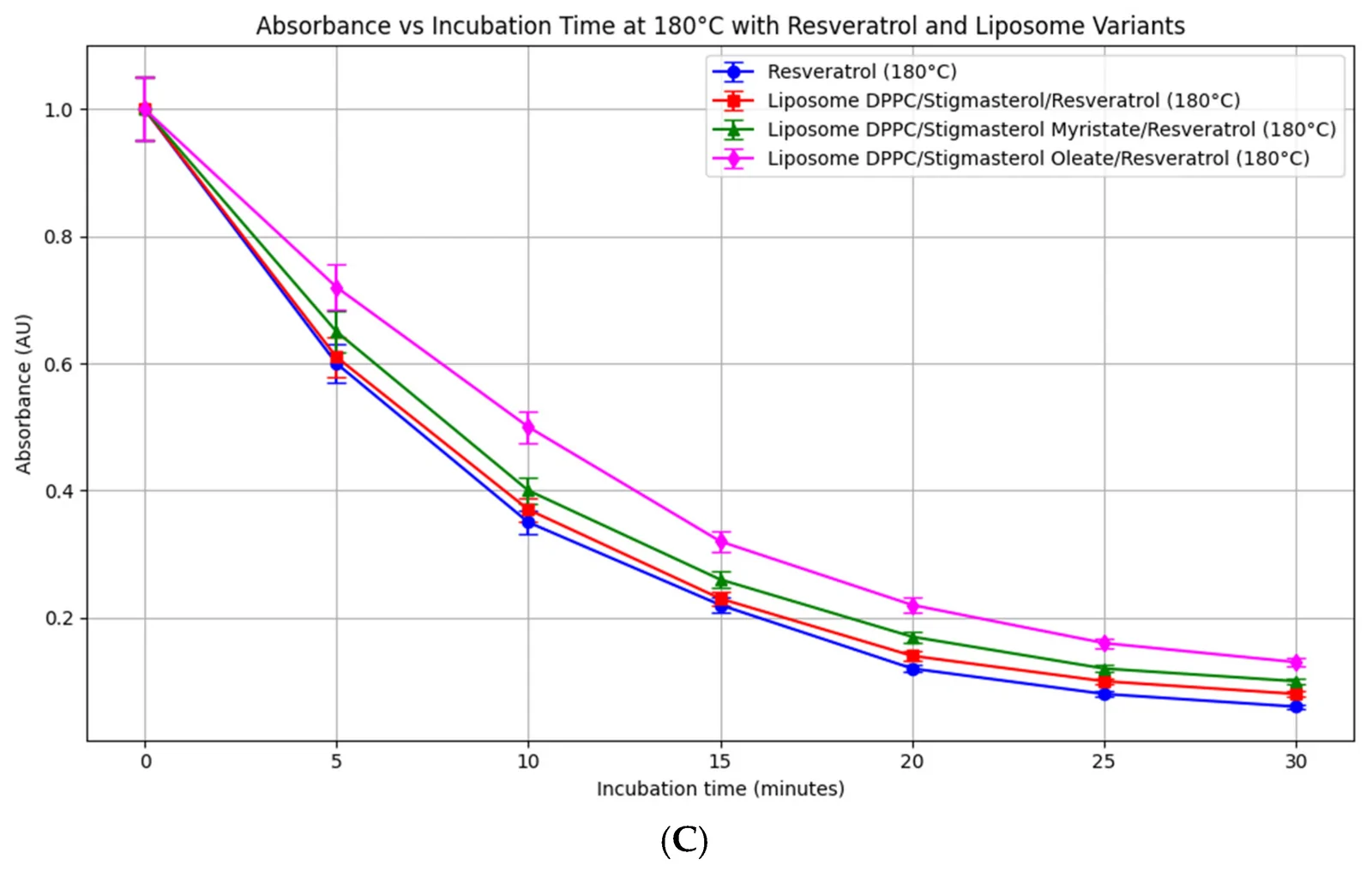
Antioxidant capacity of liposomes containing free stigmasterol (ST + RES) and its esters with myristic acid (ME + RES) and oleic acid (OE + RES), enriched with resveratrol, stored at 0 °C (**A**), 60 °C (**B**), and 180 °C (**C**).

The liposomes were added to samples containing an alcoholic solution of DPPH**•**. After measuring the absorbance, a decrease was observed in all samples. This effect is attributed to the fact that the compounds present in the samples act as strong antioxidants, and through synergistic interactions, they interrupt the radical reaction by donating hydrogen atoms or electrons, leading to the formation of more stable compounds. The DPPH**•** radical caused a reduction in absorbance in all the samples. The DPPH**•** radical scavenging ability of the samples is depending on the reactions among these compounds and other biologically active components with antioxidant properties, reactions which may happen even before liposome extraction [50]. Antioxidant protection also depends on the method and temperature of sample preparation and storage, as shown by the results in Figure 6 [51]. Classical liposomes are mainly composed of phospholipids, which may exhibit some weak antioxidant properties. Phospholipids, especially those containing unsaturated fatty acids, can react with free radicals, but their antioxidant capacity is more limited than that of resveratrol. Figure 7 shows the ability at different temperatures of liposomes containing free stigmasterol (ST + RES) and its esters with myristic acid (ME + RES) and oleic acid (OE + RES), enriched with resveratrol, to scavenge 1,1-diphenyl-2-picrylhydrazyl free radical.

Liposomes with resveratrol were added to the samples containing an alcoholic solution of DPPH**•**. Once absorbance was measured, a decrease was noted for all samples; this results from the fact that resveratrol, which acts as a strong antioxidant, interrupts the radical reaction by donating hydrogen atoms or electrons, leading to the formation of more stable compounds [52]. Stigmasterol incorporates itself into cell membranes, stabilizing their structure and reducing susceptibility to oxidative damage, for example, from hydroxyl radicals (**•**OH) and lipid peroxides. The combination of resveratrol and stigmasterol allows for effective neutralization of various types of free radicals and reactive oxygen species (ROS), both at cell membranes (stigmasterol) and intracellularly (resveratrol). Resveratrol functions in both aqueous and lipid phases, enhancing the protection of cell membranes and mitochondria from oxidative stress [39]. As a result, cells are more resistant to oxidative damage, which is particularly important for cardiovascular protection. Resveratrol improves vascular function and reduces the risk of atherosclerosis, while stigmasterol lowers LDL cholesterol levels [39,40,41,42]. After donating an electron, resveratrol can be regenerated by stigmasterol, prolonging its antioxidant activity. Stigmasterol prevents the oxidation of membrane lipids, which could otherwise reduce resveratrol’s effectiveness [40,41,42,43,44,45,46,47]. This creates a regeneration cascade that enhances the durability of both antioxidants’ action. Resveratrol activates sirtuins (SIRT1), antioxidant enzymes, and the body’s defense pathways, increasing the production of glutathione, catalase, and superoxide dismutase (SOD). Stigmasterol, on the other hand, inhibits proinflammatory cytokines (such as TNF-α and IL-6) and reduces oxidative stress through its effect on NF-κB, further limiting oxidative damage [53,54]. Stigmasterol esters with myristic and oleic acids also contribute to increased hydrophobicity of the complex, which enhances membrane protection and stability in lipid environments. Studies of the dependence of absorbance on inhibition time have also confirmed that higher temperatures reduce the antioxidant capacity of liposomes containing resveratrol. The percentage inhibition of the ST + RES 0 °C liposome differed from that of OE + RES 0 °C and ME + RES 0 °C on account of the esterification process, which blocks the hydroxyl group. Other DPPH**•**-quenching mechanisms may also be involved.

#### 2.4.3. Principal Component Analysis

Principal component analysis (PCA) was conducted to assess the variability among liposomes containing mixtures of resveratrol with stigmasterol (ST + RES) and two type of stigmasterol and fatty acid with esters (stigmasteryl myristate (ME + RES), and stigmasteryl oleate (OE + RES)) and thermal treatment.

**Figure 8:**
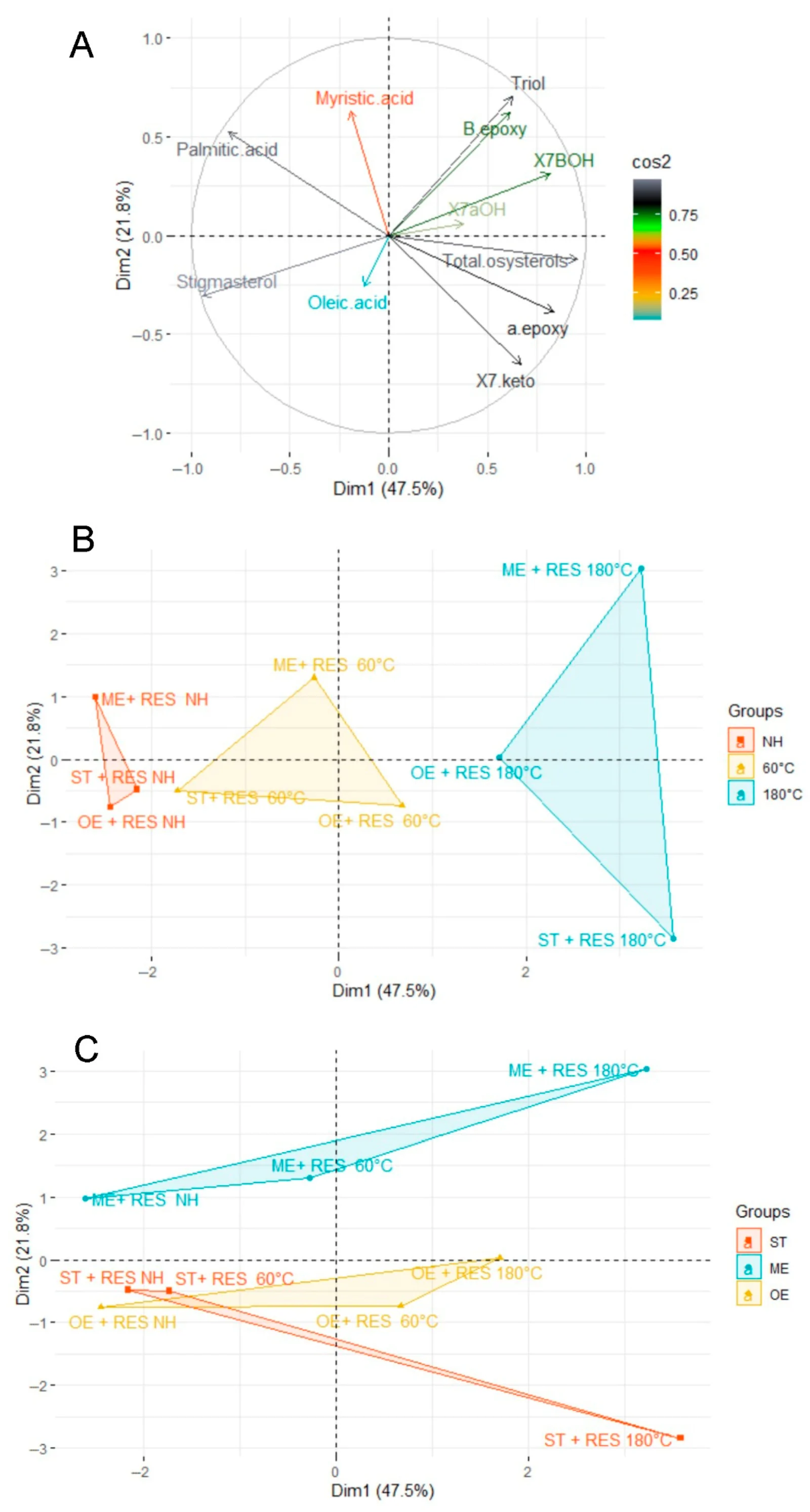
Principal Component Analysis (PCA) of the loadings plot (**A**) and the score plot (**B**,**C**) of data from liposomes with a mixture of resveratrol and stigmasterol (ST + RES), resveratrol and stigmasteryl myristate (ME + RES), and resveratrol and stigmasteryl oleate (OE + RES), subjected to three thermal treatments: not heated (NH), 60 °C, and 180 °C.

The first two principal components explained 69.3% of the total variance, with Dim1 accounting for 47.5% and Dim2 for 21.8% (Figure 8).

As can be seen from the loading plot (Figure 8), factor 1 was strongly positively correlated with total content of oxysterol (r = 0.95), α-epoxy (r = 0.83), 7β-OH (r = 0.81), 7-keto (r = 0.67), triol (r = 0.63), and β-epoxysterol (r = 0.62). It was also negatively correlated with content of stigmasterol (r = −0.94) and palmitic acid (r = −0.81). These patterns suggest that factor 1 reflects the extent of oxidative degradation, differentiating samples with high oxysterol content and low levels of native phytosterols and fatty acids. Factor 2 was positive correlated mainly with sitosterol triol (r = 0.70), myristic acid (r = 0.63), and β-epoxy sterol content (r = 0.62), and negative correlated with 7-keto (r = −0.65) and α-epoxy content (r = −0.39). This data indicates a possible association with specific oxidative transformation pathways or structural effects of esterified fatty acids.

The PCA score plots showed distinct differences in the distribution of samples depending on temperature (Figure 8B) and the type of compound used (free stigmasterol or its ester with a fatty acid) (Figure 8C). Regarding the effect of temperature, the nonheated (NH) samples clustered on the negative side of the first principal component (Dim1), indicating a low level of oxidative degradation and a high level of free stigmasterol or its esterified forms. Samples held at 60 °C were located near the center of the PCA space, reflecting moderate chemical transformation. In contrast, samples treated at 180 °C shifted toward the positive values along Dim1, corresponding to an advanced degree of oxidation and the accumulation of oxyphytosterol products. When considering the type of compound incorporated into liposomes (free or esterified stigmasterol), samples containing free stigmasterol (ST) exhibited the most pronounced shift toward positive Dim1 values after being held to 180 °C, indicating extensive oxidative degradation. Samples containing the ester of stigmasterol and myristic acid (ME) showed a similar but less intense shift along Dim1, accompanied by an additional displacement along Dim2, which may indicate the formation of specific oxidation products such as epoxysterols. Samples containing the ester of stigmasterol and oleic acid (OE) remained near the origin of the PCA space under all thermal conditions, suggesting enhanced resistance to thermal oxidation, likely attributable to the presence of the oleic acid moiety.

In summary, PCA confirmed that both the applied temperature and the chemical structure of the compound incorporated into the liposomes affected the oxidative stability of the samples. The first principal component (Dim1) represented the oxidative degradation gradient, while the second component (Dim2) reflected differences in the types of oxidation products formed. These findings indicate that esterification of stigmasterol with fatty acids, especially oleic acid, significantly improves resistance to thermal oxidation. The results have potential applications in the development of sterol-based functional ingredients with enhanced oxidative stability for use in thermally processed foods.

## 3. Materials and Methods

### 3.1. Materials

The stigmasterol (≥95%), myristic acid (≥98.0%), oleic acid (≥99%), and resveratrol (≥98.0%), with all the solvents, sodium hydroxide, anhydrous pyridine, the catalysts dicyclohexylcarbodiimide (DCC) and 4-dimethylaminopyridine (DMAP), high-purity silica gel 70–230 mesh, standards of cholesteryl oleate (98%), 5α-cholestane, 19-hydroxy-cholesterol, and heptadecanoic acid methyl ester were purchased from Sigma-Aldrich (St. Louis, MO, USA). The internal standard 19-hydroxycholesterol was purchased from Steraloids (Newport, RI, USA). The silylation mixture of BSTFA [N,O-bis(trimethylsilyl)trifluoroacetamide] with 1% TMCS (trimethylchlorosilane) was obtained from Fluka Chemie (Buchs, Switzerland), while the SEP-PAK amino cartridges were sourced from Waters Corporation (Milford, MA, USA). Dipalmitoylphosphatidylcholine (DPPC) was purchased from Avanti Polar Lipids (Birmingham, AL, USA).

### 3.2. Esterification

Stigmasteryl esters were prepared through an esterification reaction involving myristic and oleic acids. Stigmasterol was dissolved in dichloromethane at a concentration of 10 mg/mL. In this process, stigmasterol was first dissolved in dichloromethane. The catalysts (500 mg of DCC and 15 mg of DMAP) were then introduced along with the selected fatty acids. The reaction was carried out in the absence of light, under an argon atmosphere, and was maintained at room temperature for 24 h. Upon completion, distilled water was added to the reaction mixture, which was subsequently subjected to liquid–liquid extraction. The organic (lower) layer was isolated and purified by repeating the extraction step three times. Following solvent evaporation, the residue was dissolved in hexane. The resulting esters were further purified by column chromatography using silica gel. Their identity and purity were assessed via thin-layer chromatography (TLC) frim Sigma-Aldrich (St. Louis, MO, USA), using cholesteryl ester as a reference. Final verification of ester quality was conducted using gas chromatography–mass spectrometry (GC–MS) from Agilent Technologies (Santa Clara, CA, USA) [54].

### 3.3. Preparation of Liposomes

Liposomes were obtained following the protocol outlined by Cieślik-Boczula [55], with a key modification involving the incorporation of resveratrol. A mixture of organic solvents (chloroform/methanol, 2:1 *v*/*v*) in a volume of 10 mL was added, after which the solvents were evaporated to form a thin lipid film. Subsequently, 1 mL of buffer solution was introduced to the resulting lipid film at a temperature 10 °C above the gel–liquid–crystalline phase transition point. The final phospholipid concentration in the phosphate buffer was adjusted to between 10 and 30 mg/mL. The lipid dispersion was then sonicated using a probe-tip sonicator (Sonics & Materials, Inc. (Newtown, CT, USA) operating at 40% amplitude for 5 min in pulsed mode (10 son/10 s off) in an ice bath to prevent overheating. Sonication was performed on a 10 mL sample volume using a 3 mm-diameter probe tip (effective surface area ~0.07 cm^2^), which delivered approximately 80–90 J of acoustic energy to the dispersion. The resveratrol and phytosterols together with DPPC (DPPC: sterol: resveratrol = 70: 30: 5 (molar ratio)) were dissolved in chloroform/methanol (2:1 *v*/*v*) and processed together following the liposome preparation procedure described above. The obtained liposomes were then stored at 4 °C until further analysis for no longer than 24 h. Phosphate-buffered saline (PBS, 10 mM) was used as the hydration medium for liposome preparation. The final pH of all liposomal formulations was measured immediately after preparation and ranged from 7.0 to 7.1, regardless of the sterol type (ST, ME, OE) or the presence of resveratrol [55,56,57,58,59]. Lyophilized liposomes were Please confirm whether the overlapping content in this figure affects scientific understanding and if it does, please revise it. used in any physicochemical analyses (zeta potential, hydrodynamic diameter, DSC, TEM) or in thermal stability experiments. Lyophilization was applied exclusively to samples intended for chemical composition analysis, including the determination of resveratrol, stigmasterol, and oxyphytosterols (SOPs), as well as lipid and sterol extraction. All measurements of physical stability were performed using fresh, non-lyophilized liposomal dispersions.

### 3.4. Freeze-Drying of Liposomes

The liposomes were dried using an Alpha 2-4 LD freeze-dryer (Christ, Osterode am Harz, Germany) with a freezing temperature of −18 °C and a drying temperature of −20 °C for 20 h, followed by desiccation at 5 °C for 4 h, as described for liposomes containing bioactive lipophilic compounds [30,57]

### 3.5. Thermal Stability

The sample-heating procedure was carried out separately, prior to the other physicochemical analyses. The liposomal samples were:

- heated in a laboratory incubator (BINDER GmbH (Tuttlingen, Germany),
- under atmospheric conditions (air),
- in tightly sealed glass vials,
- for 8 h at temperatures of 60 °C or 180 °C. The purpose of this step was to obtain samples after controlled heating, which were subsequently analyzed (zeta potential, hydrodynamic diameter, SOPs, etc.). DSC was used exclusively for analyzing the phase transitions of DPPC and was not employed as the heating device for the thermal stability test. The liposomes were subjected to heating at 60 °C and 180 °C to assess their thermal stability under conditions simulating storage and frying. The exposure time to elevated temperature was eight hours. After heating, the ampoules containing the samples were tightly sealed and cooled to room temperature. Each heating procedure was carried out in duplicate. The temperatures applied in this study (60 °C and 180 °C) were chosen according to established models of lipid and phytosterol thermal degradation used in food science. The temperature of 60 °C corresponds to accelerated storage conditions (Schaal test), commonly applied to assess oxidative stability of lipids, sterols, and liposomal systems under simulated shelf-life conditions. This approach has also been used in our previous work on stigmasterol liposomes [30]. In contrast, the temperature of 180 °C reflects typical high-temperature food processing, such as frying, during which phytosterols undergo rapid thermal oxidation. The relevance of this temperature range is supported by earlier research on sterol degradation under culinary conditions [34]. Together, these two-temperature points model both storage-related and culinary thermal exposure scenarios relevant for phytosterols present in food matrices.

### 3.6. Phase Transition Analysis

Phase transition measurements of liposomes were performed by differential scanning calorimetry (DSC) with a DSC 7 from PerkinElmer, Inc. (Norwalk, CT, USA) equipped with an Intracooler II and Pyris Software 10.1- under nitrogen atmosphere (99.99% purity). The DSC calorimeter was calibrated using indium (m.p. = 156.6 °C, ∆H_f_ = 28.45 J/g) and n-dodecane (m.p. = −9.65 °C, ∆H_f_ = 216.73 J/g). Samples of DPPC liposomes (approximately 30 mg) were weighed into aluminum pans of 50 μL from PerkinElmer, Inc. (Norwalk, CT, USA No. B016-9321) and hermetically sealed. The reference sample contained distilled water. The sample pan was placed in the calorimeter and isothermally held at 10 °C for 5 min, then heated to 55 °C with scanning rate 2 °C/min. Two replicates were analyzed for each sample. The parameters of onset temperature (T_on_), peak temperature (T_p_), width at half height (ΔT_1/2_) and enthalpy ∆H (J/g), determined as the area limited by the curve and the baseline, were analyzed from the DSC melting curve. Additionally response ratio was calculated by dividing the peak height (mW) per ΔT_1/2_ [28,29].

### 3.7. Zeta Potential and Hydrodynamic Diameter of the Liposomes

Dynamic light scattering (DLS) is a commonly used technique for determining the size of particles undergoing Brownian motion in a liquid. To assess the hydrodynamic diameter of liposomes and their zeta potential, a Zetasizer Nano ZS-90 device (Malvern, UK) was employed. Samples were diluted tenfold with ultrapure water and immediately placed in the spectrometer following dilution. The results are presented as mean values ± standard deviations. Zeta potential measurements were performed using the electrophoretic light scattering (ELS) technique. All analyses were conducted in disposable folded capillary cells (DTS1070, Malvern Panalytical Ltd., Malvern, UK), at a controlled temperature of 25 ± 0.1 °C. Phosphate-buffered saline (PBS, 10 mM) was used as the measurement medium. The Smoluchowski model was applied to calculate electrophoretic mobility and zeta potential. Each analysis was based on three independent measurements, and the instrument was automatically calibrated and optimized according to the manufacturer’s procedure. For particle size analysis, the hydrodynamic diameter was determined using the same instrument in backscatter mode (173°). Samples were diluted in ultrapure water, and each measurement consisted of at least 15 runs [30,31].

### 3.8. Phytosterols

Stigmasterol concentrations were determined following the protocols outlined in the AOCS Official Methods Ch 6–91 (2009) and Cg 5–97 (2009) [60,61]. To begin, the samples underwent saponification using 2 M methanolic potassium hydroxide for eighteen hours. Subsequently, phytosterols were extracted with a solvent mixture of hexane and methyl tert-butyl ether (1:1, *v*/*v*). The solvent was then evaporated, and the resulting residues were derivatized with a silylating agent (BSTFA + 1% TMCS). Separation was performed on a gas chromatograph 7820A (Agilent Technologies, Santa Clara, CA, USA) coupled with a flame ionization detector (FID), using a DB-35MS capillary column (25 m × 0.20 mm, 0.33 μm; J&W Scientific, Folsom, CA, USA). Samples of 0.5 μL were injected in splitless mode. The oven temperature program began at 280 °C (where it was held for 20 min), then increased to 290 °C at 0.7 °C/min (held for 5 min), followed by a ramp to 320 °C at 30 °C/min, with a final hold of 5 min. Hydrogen served as the carrier gas at a flow rate of 2 mL/min. Stigmasterol was identified by comparing retention times with those of known standards prepared through identical derivatization. 5α-cholestane served as the internal standard, and stigmasterol levels were confirmed by alignment with reference compounds [33].

### 3.9. Fatty Acids

The fatty acid composition was determined by gas chromatography according to the AOCS Official Method Ce 1k-07 (2007) [58], using an 8890 GC system (Agilent Technologies, Santa Clara, CA, USA) equipped with a flame ionization detector (FID). Separation was performed on an SP™-2560 capillary column (100 m × 0.25 mm × 0.2 μm; Supelco, Bellefonte, PA, USA). Hydrogen was used as the carrier gas at a flow rate of 1.5 mL/min. A 0.5 μL sample was injected for each analysis. The oven temperature program started at 60 °C (held for 1 min), followed by an increase of 6 °C/min to 220 °C. The injector and detector temperatures were set at 240 °C. A commercial FAME mix Supelco 37 Component FAME Mix (Supelco, Sigma-Aldrich, Bellefonte, PA, USA) was used to identify fatty acids, and quantification was carried out using an internal standard (C17:0) [62].

### 3.10. Stigmasterol Oxidation Products (SOP)

To identify derivatives of oxidized phytosterols, the procedure outlined by Rudzińska et al. [34,35] was employed. Lipids were first extracted via the Folch method and supplemented with 0.006% BHT to prevent oxidation. The extracted lipids were then subjected to transesterification using sodium methoxide. Following chloroform extraction, the mixture was separated using SEP-PAK NH_2_ cartridges. The resulting fractions were silylated and analyzed with a gas chromatograph 7820A (Agilent Technologies, Santa Clara, CA, USA) equipped with a DB-35MS capillary column (25 m × 0.20 mm × 0.33 μm; J&W Scientific (Agilent Technologies, Folsom, CA, USA). The oven temperature was programmed to rise from 50 °C to 270 °C at a rate of 25 °C/min, then from 270 °C to 290 °C at 1 °C/min, and was held at 290 °C for 95 min. For quantification, 19-hydroxy-cholesterol served as the internal reference. Compounds were identified by comparing sample retention times with those of reference standards prepared under identical derivatization conditions. The content of stigmasterol oxidation products (SOPs) was expressed as milligrams of SOP per gram of stigmasterol (mg/g) [36,37].

### 3.11. Antioxidant Activity of Liposomes

The antioxidant activity of the liposomes was assessed using a modified method originally developed by Brand-Williams et al. [60], using DPPH**•** (1,1-diphenyl-2-picrylhydrazyl) as a free radical. Absorbance was measured at a wavelength of λ = 517 nm. A DPPH alcohol solution was prepared by dissolving 2.8 mg of DPPH**•** (M = 394.32 g/mol) in 100 mL of methanol; its absorbance was then recorded as a control (A_0_ = 1). The solution was stored in a dark environment. To prepare both the resveratrol-containing control samples and the liposomal samples, 1 mg of liposomes was extracted with methanol, followed by vigorous mixing for approximately five minutes. The extract was then stored in the dark. A volume of 2 mL of the prepared extract was added to 3 mL of DPPH solution. The mixture was incubated in a water bath at 37 °C for thirty minutes. The absorbance was monitored using a TCC-240A UV-Vis spectrophotometer (Shimadzu, Kyoto, Japan) for thirty minutes from the initiation of the reaction. The results were expressed as the percentage of free radical scavenging, calculated using the following equation:

% Inhibition = 100 × (A_0_ − A_1_)/A_0_ (1)

where A_0_ is the absorbance of the DPPH**•** solution after thirty minutes without the addition of the sample, and A_1_ is the absorbance of the DPPH**•** solution after 30 min in the presence of the sample [63].

### 3.12. Statistical Analysis

All experiments and analyses were carried out in triplicate, and the results were expressed as mean values accompanied by standard deviations (± SD). Statistical computations were conducted using Statistica software, version 13.3 (StatSoft, Tulsa, OK, USA). The significance of differences among groups was assessed using one-way analysis of variance (ANOVA).

## 4. Conclusions

The DPPC liposomes enriched with resveratrol were formulated to contain either free stigmasterol, stigmasteryl myristate, or stigmasteryl oleate. Their thermal stability was assessed by subjecting them to temperatures of 60 °C and 180 °C, simulating conditions of accelerated test storage and frying, respectively. Among the formulations, the ST + RES liposomes were the smallest in size, whereas the ME + RES and OE + RES liposomes were larger and similar in size to each other. Notable shifts were also observed in zeta potential and hydrodynamic diameter, indicating that both resveratrol and stigmasterol esters contributed to membrane stabilization at 60 °C. However, this protective effect was inadequate under the more extreme conditions of 180 °C. The extent of compound degradation within the resveratrol-loaded liposomes appeared to be influenced by the structural form of the encapsulated phytosterol. At 60 °C, liposomes containing free stigmasterol showed the highest thermal resilience. When heated to 180 °C, free stigmasterol degraded most rapidly, whereas stigmasteryl oleate displayed the greatest thermal stability. Moreover, the persistently elevated levels of oxyphytosterols detected in the samples treated at 180 °C, especially in the ST + RES and ME + RES formulations, raise concerns about oxidative degradation. The findings suggest that in food products not exposed to high temperatures, resveratrol-loaded liposomes with free phytosterols offer better performance than their esterified counterparts. In contrast, for fried or thermally processed foods, combining resveratrol with phytosterol esters, particularly those derived from oleic acid, appears to offer better protection against oxidative damage. Liposomes containing resveratrol exhibit strong antioxidant properties, which contributed to the increased stability of the liposomes. In conclusion, the use of such innovative carriers may significantly enhance the stability, bioavailability, and epithelial absorption of phytosterols in the gastrointestinal tract. The combined health-promoting effects of resveratrol and phytosterols may have considerable and unprecedented significance for human health.

## Figures and Tables

**Table 1:** Thermotropic parameters calculated from DSC phase transition curves for resveratrol-enriched DPPC liposomes (RES) and resveratrol-enriched DPPC liposomes with free stigmasterol (ST), stigmasteryl oleate (OE), and stigmasteryl myristate (ME).

Type of DPPC Liposomes	Onset Temperature T_on_ (°C)	Peak Temperature T_p_ (°C)	Enthalpy ΔH (J/g)	Width at Half Height ΔT_1/2_ (°C)	Response Ratio (mW/°C)
RES	40.17 ± 0.01	41.39 ± 0.06	44.24 ± 0.44	1.24 ± 0.08	0.75 ± 0.15
ST	nd	nd	nd	nd	nd
OE	36.22 ± 0.21	39.46 ± 0.62	32.43 ± 0.94	2.13 ± 0.10	0.11 ± 0.01
ME	37.77 ± 0.06	42.35 ± 0.17	28.71 ± 0.23	4.51 ± 0.14	0.03± 0.01

nd—not detected, mean ± standard deviation (*n* = 2).

**Table 2:** Potential zeta and hydrodynamic diameter of liposomes loaded with resveratrol and stigmasterol (ST + RES), stigmasterol myristate (ME + RES), and stigmasterol oleate (OE + RES) before and after being held at 60 °C and 180 °C for eight hours.

Sample	Unheated 25 °C	Held at 60 °C	Held at 180 °C
Zeta potential (mV)			
ST + RES	2.9 ± 0.1 ^c^	−15.0 ± 0.4 ^b^	−41.2 ± 1.2 ^a^
ME + RES	3.1 ± 0.1 ^b^	8.3 ± 0.4 ^c^	−38.5 ± 1.9 ^a^
OE + RES	2.0 ± 0.1 ^b^	4.7 ± 0.2 ^c^	−33.5 ± 0.3 ^a^
Hydrodynamic diameter (nm)			
ST + RES	214.2 ± 3.5 ^a^	336.6 ± 10.8 ^b^	556.6 ± 53.2 ^c^
ME + RES	644 ± 93.7 ^b^	294.3 ± 25.1 ^a^	244.8 ± 12.2 ^a^
OE + RES	945 ± 113.3 ^b^	276.6 ± 17.2 ^a^	188.6 ± 4.4 ^a^

^a^, ^b^, ^c^—Means with different letters differ significantly (*p* < 0.05). All zeta potential (ZP) and hydrodynamic diameter measurements were performed at 25 ± 0.1 °C.

## Data Availability

The original contributions presented in the study are included in the article; further inquiries may be directed to the corresponding author.

## Abbreviations

DPPC: dipalmitoyl phosphatidylcholine; RES: resveratrol; Liposomes RES: liposomes encapsulated with resveratrol; ST: liposomes encapsulated with free stigmasterol; ST + RES: liposomes encapsulated with resveratrol and free stigmasterol; ME: liposomes encapsulated with stigmasterol myristate; ME + RES: liposomes encapsulated with resveratrol and stigmasterol myristate; OE: liposomes encapsulated with resveratrol and stigmasterol oleate; OE + RES: liposomes encapsulated with stigmasterol oleate; BSTFA: N,O-bis(trimethylsilyl)trifluoroacetamide; MTBE: tert-butyl methyl ether); 0 °C: (unheated liposomes); 60 °C: (liposomes treated at 60 °C); 180 °C: (Liposomes treated at 180 °C); SOP: Stigmasterol oxidation products; 7αOHSt: 7α-hydroxystigmasterol; 7βOHSt: 7β-hydroxystigmasterol; α-epoxySt: 5α,6α-epoxystigmasterol; β-epoxySt: 5β,6β-epoxystigmasterol; 7ketoSt: 7ketostigmasterol; triolSt: 3β,5α,6β-trihydroxystigmasterol; DPPH**•**: (1,1-diphenyl-2-picrylhydrazylu).
