# Correction: Igielska-Kalwat et al. Changes in Resveratrol Containing Phytosterol Liposomes During Model Heating. *Molecules* 2025, *30*, 4645

**DOI:** 10.3390/molecules31152692

**Published:** 2026-08-03

**Authors:** Joanna Igielska-Kalwat, Magdalena Rudzińska, Anna Grygier, Dominik Kmiecik, Katarzyna Cieślik-Boczula, Jolanta Tomaszewska-Gras

**Affiliations:** 1 Faculty of Food Science and Nutrition, Poznań University of Life Sciences, Wojska Polskiego 28, 60-637 Poznań, Poland; anna.grygier@up.poznan.pl (A.G.); dominik.kmiecik@up.poznan.pl (D.K.); jolanta.tomaszewska-gras@up.poznan.pl (J.T.-G.); 2 Faculty of Chemistry, University of Wroclaw, F. Joliot-Curie 14, 50-383 Wrocław, Poland; katarzyna.cieslik-boczula@uwr.edu.pl

**Missing acknowledgement**

In the original publication [1], the following funder information was not included: **This study was financed by the National Science Centre Poland, grant number 2021/43/B/NZ9/00345.** The authors provide the following clarification: “The article was prepared based on results obtained within the framework of a funded research project. In order to properly report and settle the grant, we are required to provide a list of all publications resulting from the project. Unfortunately, the National Science Centre (NCN) cannot accept this publication as an outcome of the grant because the funding acknowledgement sentence that I mentioned is missing from the published article. Therefore, adding this information is very important for us and for the correct reporting of the project results”. The authors state that the scientific conclusions are unaffected. This correction was approved by the Academic Editor. The original publication has also been updated.
